# Configurational analysis of conditions influencing customers’ channel switching intention in omnichannel retailing: a fuzzy-set analysis

**DOI:** 10.1007/s11135-023-01633-8

**Published:** 2023-03-02

**Authors:** Anh Thi Van Nguyen, Alrence S. Halibas, Robert McClelland, Nguyen Hoang Thuan

**Affiliations:** RMIT Vietnam, 702 Nguyen Van Linh Street, District 7, Ho Chi Minh City, Vietnam

**Keywords:** Channel switching behaviour, Customer experience, Configurational analysis, fsQCA, Omnichannel, Qualitative comparative analysis (QCA)

## Abstract

This research examined the interplay among personal factors, namely channel lock-in, cross-channel synergy, attribute-based decision making (ADM); environmental factors, namely others’ past switching behaviour (OPB), pressure to switch from others (PSO); and behavioural factors, namely perceived self-efficacy and perception on facilitating conditions as antecedents to customers’ channel switching intention in an omnichannel context. Drawing on the complexity theory and set theory, we applied configurational analysis using the fuzzy-set Qualitative Comparative Analysis. The result of the analysis indicated two (2) sufficient configurations that led to an intention to switch channels. Both configurations contained ADM, OPB, and PSO conditions that highlight the importance of personal factors and environmental factors needed for the presence of an intention to switch channels. However, no sufficient configurations were obtained that indicate an absence of intention to switch channels. This study challenges theoretical underpinnings by demonstrating that omnichannel channel-switching behaviours can be explained from a configurational perspective. The configurations produced by this study can serve as a basis for researchers who plan to conduct asymmetric modelling of customers' channel-switching behaviour in an omnichannel context. Finally, this paper suggests omnichannel retail strategies and management as informed by these configurations.

## Introduction

In recent years, customers have increasingly become efficient shoppers who can use multiple channels during their shopping journey. They know what product they want, when, and what channel to use for search and purchase (Crawford 2005; Frasquet and Miquel-Romero 2021). They also prefer to deal with retailers that can provide them with a convenient and seamless shopping experience across channels (Yurova et al. 2017). These omnichannel shoppers prefer various channel options during their purchase journey (Chiou et al. 2012) and use cost- and resource-effective means to search for and purchase products. Omnichannel shoppers are profitable customers since they spend more than single-channel shoppers (Sopadjieva, Dholakia, and Benjamin 2017).

Given the importance of omnichannel customers, retailers must understand how and when they use the different channels and what drives them to switch channels (Pookulangara et al. 2011). However, it is challenging to understand the complexity and nature of customer behaviour in an omnichannel retailing context, especially customers' channel-switching behaviour during their search and purchase journey (Neslin et al. 2014). This difficulty was further heightened during the Covid-19 period when online shopping rose sharply while in-store traffic declined. The situation necessitates retailers to adjust their channel strategy by shifting to an omnichannel approach to attract online customers and maximise offline operations for cash flow and customer engagement (Zhuang et al. 2020). Implementing unified management of the channels can effectively enhance the channel performance and customer experience (Nguyen, McClelland, and Thuan 2022; Verhoef, Kannan, and Inman 2015). Therefore, we are interested in exploring customer behaviour in an omnichannel retailing context to fit the new and ever-changing business environment.

Past research studies in the omnichannel retailing domain mainly examined the retailers’ perspectives rather than the customers’ perspectives (Truong, 2021). Likewise, prior research on omnichannel strategies focused on the technical perspective (Bell, Gallino, and Moreno, 2014). Nevertheless, few research studies attempted to understand the customer channel-switching experience by exploring the channel-switching causes, intention, and behaviour. For instance, (Verhoef et al. 2007, p. 130) based on the theory of reasoned actions to develop a model to “understand why customers choose different channels for search and purchase” and investigated potential strategies for managing the research shopper phenomenon. Recently, Nguyen, McClelland, and Thuan (2022) expanded the model and found five reasons for omnichannel switching behaviour. The authors further called for more studies to examine factors influencing customers' channel-switching intention. Similarly, Xu and Jackson (2019) called for more studies on channel selection intention in an omnichannel setting. These calls suggest a research gap in the domain.

This research gap can be further seen from a methodological viewpoint where most existing studies in channel switching applied symmetric modelling to generalise the outcome behaviour, notably regression analysis and structural equation modeling (SEM) (Haridasan et al. 2021). For instance, Martinelli et al. (2021) stated that attitude, subjective norms, and perceived behavioural control are significant factors behind customers' online switch decisions. Likewise, Youn et al. (2021) showed that response and self-efficacies of channel switching increase the beliefs and intentions of customers to switch to online shopping channels. In adopting symmetric methods, these studies mainly examined the independent effects of factors, yet provided little understanding of the combinatorial effects of factors. To our knowledge, few studies have adopted an asymmetric method for examining factors influencing customers' channel-switching intention and asymmetric causalities among these factors.

The methodology literature has clarified the difference between symmetric and asymmetric methods. Symmetric methods are arguably inadequate for theory construction and testing because of the asymmetric nature of data and having no test for predictive validity (Woodside 2014). They dismiss outliers that do not follow the hypothesised relationship (Ragin 1987). Armstrong (2012) also asserted that regression analysis could confuse, reduce accuracy, and cause overconfidence. Similarly, Gligor and Bozkurt (2020) claimed that the symmetric approach could not capture the complexities of customer engagement as it generalises the relationships. They maintained that asymmetric modelling, such as Qualitative Comparative Analysis (QCA), can be used as a stand-alone method because it provides sufficient explanatory power to relationships of interest (Gligor and Bozkurt 2020). Instead of examining the net effect of variables, QCA looks at the causation effects of the combinations of conditions. On the other hand, QCA has its share of shortcomings. Fainshmidt et al. (2020) reported several limitations of QCA including the complexity of interpreting the results especially when it involves a large number of causal conditions and the sensitivity of the data calibration process, among others. Nonetheless, this study applied QCA to explore the reasonable number of factors that can lead to a presence and absence of a channel-switching intention.

The main research question is, “*What configurations of antecedent personal, behavioural, and environmental factors of customer experience can explain the presence and absence of channel-switching intention in an omnichannel context?”* To address this question, we employed QCA, which is a modern research method increasingly used in marketing and customer studies, to identify combinations of causal configurations affecting customers' channel-switching intention. We believe understanding customer behaviour is complex and assert that a substantive knowledge of the complexity and configurational theories can explain channel switching behaviour. At the same time, we extend the theoretical understanding of the research shopper model of Verhoef et al. (2007) in an omnichannel context. Moreover, Pappas and Woodside (2021) suggest extending and supplementing existing papers that use variance-based methods with QCA. Hence, this paper extend Nguyen et al. (2022)’s research on experience during channel switching in omnichannel retailing.

We organised the succeeding sections of this paper as follows. Section 2 provides the theoretical and methodological background that summarises the key factors affecting channel-switching behaviour in an omnichannel context. Next, Sect. 3 presents the configuration model and research propositions. Thereafter, Sect. 4 provides the research method used in this study, including a step-by-step account of the fsQCA method, its analysis, results, and discussions. Section 5 provides the theoretical, methodological, practical, and managerial implications. Finally, Sect. 6 provides the conclusion.

## Theoretical and methodological background

Parker and Hand (2009) and Ortis and Casoli (2009) introduced the term “Omni" in the IDC’s Global Retail Insights research unit reports. “Omni” is a Latin word meaning “all” or “universal”; hence, an "omnichannel shopper” refers to the evolution of the multichannel customer who uses integrated channels simultaneously. Verhoef, Kannan, and Inman (2015) defined omnichannel management as the synergetic management of many available channels and customer touchpoints to optimise the customer experience across channels.

### Omnichannel switching behaviour literature

In the multichannel retailing context, the literature on switching behaviour has two (2) key themes: showrooming and webrooming. Showrooming is when the customers search for information about the product offline but purchase it online (Bell et al. 2014; Rapp et al. 2015; Verhoef et al. 2015; Basak et al. 2017). Contrastingly, webrooming is the practice of researching products online or on a mobile device but purchasing products offline (Aw 2020). For instance, Amazon’s stores use a showrooming approach that leverages a mobile point of sale with image recognition and augmented reality features to improve customers’ in-store shopping experience (Johnson, n.d.). Amazon’s customers can use an iPhone app to scan a product in the physical showroom. The app will search for the product on Amazon's e-commerce store, where the customer will eventually purchase it.

Showrooming and webrooming research have been sufficiently studied in multichannel and cross-channel contexts but are relatively new in the omnichannel context. Most research studies focused on how firms deploy strategies to retain customers during shopping (Verhoef, Inman, and Kannan, 2015). This paper considers the omnichannel switching behaviour when customers move freely between any channels, whether online or offline.

Previous studies examined the reasons for the phenomenon in multichannel and omnichannel contexts. Verhoef et al. (2007) developed a model to understand the reasons for channel switching in the multichannel context and investigate potential strategies for managing the “research shopper phenomenon”. Based on the theory of reasoned actions, the model outlined three (3) reasons for channel switching: low channel lock-in, cross-channel synergy, and attribute-driven decision-making. Barwitz and Maas (2018) studied customer interaction choices during the shopping journey and concluded that omnichannel channel switching was a pattern of channel choices. In the omnichannel retailing context, Nguyen et al. (2022) found two more reasons for omnichannel channel switching: the influence of social groups and perceived self-efficacy of the behaviour and proposed using social cognitive theory to develop a framework to explain the switching intention.

### Social cognitive theory

**Fig. 1:**
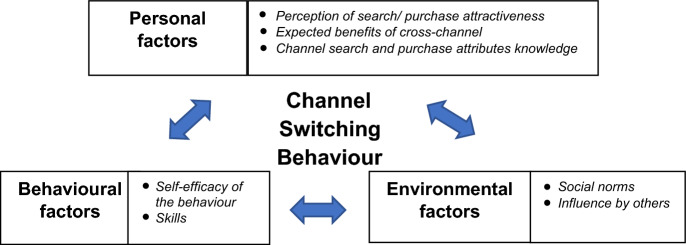
Key factors affecting channel switching behaviour (Nguyen et al. 2022)

According to Bandura (1986), social cognitive theory explains the psychology behind human thoughts, emotions, and behaviours. A person’s behaviour is determined by a mechanism of one’s behaviour, personal, and environmental factors (judgment of one’s behaviour concerning environmental circumstances) (Bandura 1991). Based on the theory and Nguyen et al. (2022), this research proposes that personal factors, behavioural factors, and environmental factors can explain omnichannel customers’ channel-switching behaviour (Fig. 1).

The framework further extends the research shopper model of Verhoef et al. (2007), which adopted the theory of reasoned actions. The authors confirmed empirically that attitudes towards different channels cause the channel-switching phenomenon (personal factors). Nguyen et al. (2022) proposed adding behavioural and environmental factors to the model using exploratory qualitative research. This research continues to examine the proposed reasons for the presence and absence of channel switching intention using quantitative research methods and QCA analysis.

### Personal factors

According to the social cognitive theory, personal factors consist of knowledge, expected outcomes, and attitudes towards a behaviour (Bandura 1991). In this research, personal factors included three main reasons for channel-switching behaviour derived from the research shopper model of Verhoef et al. (2007) and the theory of reasoned action.

If the customers perceived one channel as search attractive but a different channel as purchase attractiveness, the switching action would happen (Verhoef et al. 2007). Nguyen et al. (2022) confirmed the difference in participants' perceived search and purchase attractiveness of different online and offline channels. Furthermore, their findings showed that customers’ perception of search and purchase attractiveness in online and offline channels impacted their attitude towards channel switching, which could affect the channel switching intention and behaviour.

Similarly, switching happens when the customers believe that the benefits from one search channel would enhance another purchase channel. Verhoef et al. (2007) found a positive synergy between searching on a channel and purchasing on a different channel. In the omnichannel retailing context, Nguyen et al. (2022) found that customers switch to earn benefits from all channels.

Lastly, customers switch between search and purchase when they perceive one channel excels on search attributes and the other excels on purchase attributes. Verhoef et al. (2007) called this reason “attributed-based decision making”, which means that customers make decisions based on their knowledge of channel attributes. Through qualitative interviews, Nguyen et al. (2022) also found that omnichannel customers perceived online and offline channels with different features for search and purchase.

### Behavioural factors

According to Bandura (1991) and Nguyen et al. (2022), a person’s channel-switching behaviour is determined by the belief in self to conduct the behaviour (or self-efficacy) and his ability to complete the behaviour.

The concept of self-belief to conduct the behaviour is closely related to perceived behaviour control. According to Ajzen (1991), perceived behavioural control refers to people's perceptions of their capacity to perform a specific behaviour and the extent to which they have the prerequisite resources to achieve it. The constructs of perceived behavioural control should be separated to reflect the perceptions of external and internal forces that could influence the behaviour (Terry and O’Leary 1995). The internal force refers to the customers’ perception of their ability to perform the behaviour, which is closely related to the perceived self-efficacy concept of Bandura (1978, 1982).

In the context of omnichannel switching behaviour, customers' perception of their knowledge and experience of the product and the shopping journey is an essential factor that could influence the channel choices of customers during their journey (Chiu et al. 2011).

On the other hand, external forces include the facilitating conditions (e.g. time, facilities, and technology) needed to perform the behaviour. Customers will switch when they perceive they have the resources of time, technology, location, knowledge, and skills to carry out the switching behaviour. Studies showed that the more knowledge and experience customers have, the more likely they would switch channels, particularly showrooming (Balasubramanian et al. 2005; Verhoef et al. 2007).

### Environmental factors

The societal cognitive theory stresses that a person’s behaviour can be affected by his environmental environment. Environmental factors concern the judgment of one’s behaviour concerning environmental circumstances.

For this research, the environmental factors consist of others’ past switching behaviour (OPB) and pressure to switch from others (PSO). This means that the customers will switch between channels if they observe similar behaviour patterns from peers or when being advised by others. This means that expectations of others could influence one’s perceived intention and behaviour (Ajzen 1991), and the motivation to comply with social pressure and the references’ influence on one’s perceived behaviour.

Previous studies proposed to expand the concepts of social and descriptive norms (Armitage and Conner 2001; Ham et al. 2015; Rivis and Sheeran 2003). Descriptive norms are actual activities and behaviours of others, whereas social norms are the perceptions of others' views on how an individual should act. This paper considers social and descriptive norms, assuming that social norms can affect omnichannel customer channel-switching behaviour.

In summary, Table 1 provides the operational definitions of the constructs of this study. The constructs will serve as causal conditions to show the presence or absence of the intention outcome in the configuration model, as shown in the next section.

**Table 1:** Operational Definition of Constructs

Variable	Construct (or condition)	Definition	Theory/literature source
Personal factors	Channel lock-in (CLI)	If customers perceive one channel as search-attractive, then the same channel can be perceived as purchase attractive	Nguyen et al. (2022), Verhoef et al. (2007)
	Cross-channel synergy (CCS)	If customers perceive one channel as search or purchase attractive, then another channel can also be perceived as search or purchase attractive	Nguyen et al. (2022), Verhoef et al. (2007)
	Attribute-based decision-making (ADM)	If customers perceive that one channel excels on search attributes and others excel on purchase attributes	Nguyen et al. (2022), Verhoef et al. (2007)
Environmental Factors	Others' past switching behaviour (OPB)	Customers’ observation of other people's past switching experience	Nguyen et al. (2022)
	Pressure to switch from others (PSO)	Customers’ perception that they should switch under the expectations of others	Nguyen et al. (2022)
Behavioural factors	Perceived self-efficacy (PSE)	Customers’ perception that they can control/perform the switch	Nguyen et al. (2022)
	Perception of facilitating conditions (PFC)	Customers perceive the required resources, knowledge, and skills to conduct the switch	Nguyen et al. (2022), Taylor and Todd, (1995)
Likelihood of switching channels	Intention (INT)	Switching behaviour happens when an individual has the motivation (intention to switch)	Ajzen, (1991)

### Qualitative comparative analysis

According to Ragin (1987), QCA is an asymmetric data analysis technique that combines qualitative and quantitative approaches. The author developed QCA in the 1980s as a comparative approach to assess causation. It analyses relationships through the systematic identification of configuration membership (Leppänen et al. 2019). The method combines case-oriented and variable-oriented approaches and models multiple realities with complex antecedent configurations (Thai and Wang, 2020; Woodside 2014). There are two popular QCA methods: crisp-set QCA (csQCA) and fuzzy-set QCA (fsQCA). csQCA is exclusively based on binary factors (or conditions), whereas fsQCA is based on fuzzy-set membership scores and sub-set relations (Denk and Lehtinen 2014).

Complexity theory claims that no simple condition can produce an outcome of interest. Instead, there are combinations of causal conditions for such an outcome (Wu et al. 2016). Although QCA is not designed to provide general relationships among variables, it can give nuances, exploration, and explanation of cases (Sukhov et al. 2021). QCA was mainly used to analyse complicated social and political conditions (Berg-Schlosser et al. 2012). At present, fsQCA is increasingly used in different fields and industries, namely management (Amara et al. 2020; Cotte Poveda and Pardo Martínez, 2013; Salonen et al. 2021), innovation (Kraus et al. 2018; Leppänen et al. 2019), marketing (Phung et al. 2020), tourism (Olya and Gavilyan 2017), and mobile gaming (Pappas et al. 2019).

Configurational analysis is an emerging approach to analysing complex customer behaviour. QCA is founded on the configurational theory that describes causal complexities using the logic of set theory (Misangyi et al. 2017). Unlike a conventional variance-based analysis that produces a single optimal solution, QCA provides identifiable cases in the dataset that can verify the set propositions and offers richer contextual insights (Pappas and Woodside 2021). It uses Boolean algebra to find combinations of factors and examine their relationships to explain the critical conditions contributing to an outcome (Greckhamer, Misangyi, and Fiss, 2013; Zhao and Yan 2020). Contrastingly, regression analysis focuses on the net effects a variable has on an outcome of interest, while QCA focuses on combinations of causal conditions that lead to an outcome (Elliot 2013). Several research studies argued that the resulting configurations in QCA can generate new knowledge. According to Pappas and Woodside (2021), QCA provides theory construction from case-based outcomes. Greckhamer et al. (2013) confirm that QCA is helpful for theory construction, elaboration, and testing. According to Duşa and Thiem (2015), the core mechanism of QCA is "to reduce an exhaustive output function which represents a formal description of regularity patterns to a minimally complex equivalent that still preserves all the essential properties of the original representation of the function' (p. 93). This reduction process is called Boolean minimisation, which uses the Quine-Mc Cluskey algorithm to find the minimum combinations of causal conditions for an outcome.

Salonen et al. (2021) described fsQCA as a recognised research method suitable for causal explanations of the antecedents. However, Finn (2022) critiqued that QCA can only provide the "what" but not the "how" and "why". Hence, he suggested that QCA should complement other methods. So far, fsQCA has been coupled with SEM and other research methods to understand the complexities of customer behaviour. For example, SEM and fsQCA appeared in the study of Urueña and Hidalgo (2016) to explore antecedents of customer loyalty following complaint behaviour in B2C e-commerce. Yang (2021) also used SEM and fsQCA to validate the significance of social capital on customers' purchase intention in social commerce. Likewise, Gligor and Bozkurt (2020) used Multiple Regression Analysis and fsQCA to explore factors that lead customers to engage with brands. Nevertheless, QCA is suitable as a stand-alone method because of its sufficient explanatory power (Gligor and Bozkurt 2020).

## Configuration model and research propositions

### Configuration model

In terms of theory-building, QCA needs to be better established as compared to correlation studies; thus, there is a need to develop configurational arguments to justify the causal conditions used to explain the outcome (Greckhamer et al. 2018). In this regard, we build a configuration model to explain the channel switching intention outcome.

**Fig. 2:**
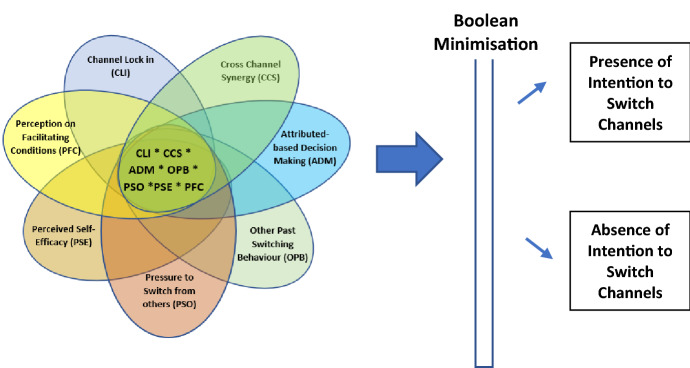
Venn diagram of the Configuration Model

Figure 2 shows a set-theoretic configuration model that conceptualises the constructs and illustrates how they have multiple asymmetric relations. The presence or absence of intention to switch channels can occur with various combinations of constructs, namely channel lock-in (CLI), cross-channel synergy (CCS), attribute-based decision-making (ADM), others' past switching behaviour (OPB), pressure to switch from others (PSO), perceived self-efficacy (PSE), and perception of facilitating conditions (PFC). The outcomes of interest are processed using Boolean minimisation.

### Research proposition

The research proposition is derived from the configuration model to help understand the complexity of the intention outcome. 

*General proposition*: The combinations of CLI, CCS, ADM, OPB, PSO, PSE, and PFC conditions lead to the presence and absence of channel-switching intention.

Conjunctural causation combines multiple causal conditions (Berg-Schlosser et al. 2012). In general, this paper includes different constructs from the literature and identified combinations of these constructs that lead to a channel-switching intention. Furthermore, QCA treats an outcome as configurations of interconnected structures, not individual entities (Pappas and Woodside 2021). Therefore, we treat the constructs as causal conditions that lead to a channel-switching intention.

There are limited configurational studies that can support this proposition. The configurational study of Chou et al. (2016) demonstrates a sufficient configuration involving factors related to perceived risk and switching barriers associated with an online store. These factors include attitude toward switching, subjective norms, switching cost, past switching behaviour, and variety seeking, which contribute to a cross-channel free-riding intention. Furthermore, Arora et al. (2020) find that showroomers are driven by perceived showrooming value which combines in-store search value and online purchase value and showrooming self-efficacy based on the stimulus–organism–response model.

There are several ways in which different configurations of CLI, CCS, ADM, OPB, PSO, PSE, and PFC conditions relate to a specific channel-switching intention. The principle of equifinality supposes that there is no single explanation for particular occurrences, but several reasons exist for an outcome (Schneider and Wagemann 2012). In applying this principle, we supposed multiple configurations could lead to the same channel-switching intention.

Different configurations of CLI, CCS, ADM, OPB, PSO, PSE, and PFC conditions can lead to the presence, at the same time, absence of a channel switching intention. This describes causal symmetry, which means that the presence and absence of the outcome intention produce different causal explanations (Berg-Schlosser et al. 2009). The configurations in the absence of the intention outcome are unique and are not mirror opposites of the configurations in the presence of the intention outcome (Woodside 2018).

Veríssimo (2016) suggests that the analysis of the causal conditions should include the presence and absence of the intention outcome. Thus, configurations that involve combinations of causal conditions will produce the outcome's presence (INT) and absence (~ INT). The following Boolean functions are illustrated in formulas 1 and 2 as follows:

1 $${\text{INT}} = {\varvec{f}}\left( {{\text{CLI}},\;{\text{CCS}},\;{\text{ADM}},\;{\text{OPB}},\;{\text{PSO}},\;{\text{PSE}},\;{\text{PFC}}} \right)$$

2 $$\sim {\text{INT}} = {\varvec{f}}\left( {{\text{CLI}},\;{\text{CCS}},\;{\text{ADM}},\;{\text{OPB}},\;{\text{PSO}},\;{\text{PSE}},\;{\text{PFC}}} \right)$$

where ~ symbolises negation

To our knowledge, no known study reports the combinations of causal conditions that explain the absence of intention to switch channels. Hence, this study will supplement this literature gap.

## Methodology

### Sample and data collection

This research was carried out among millennial customers of omnichannel electronic goods retailers in Vietnam. Despite being an emerging economy, omnichannel retailing is proliferating in the country. Reports have shown that companies are increasing investment in the omnichannel experience from 20% to 80% and more (PwC 2020). In Vietnam, there has been a notable increase in companies catching up with the trend. The Mobile World Investment Corporation—the leading mobile phone and customer electronics retailer in Vietnam with 4000 stores—launched its omnichannel strategy in 2011 (Hoa 2017). After that, the FPT Digital Retail Joint Stock Company, one of the top four retailers of customer electronics goods, followed the trend by adopting omnichannel three years later (Chungta 2017). By 2022, about 76% of businesses in Vietnam will consider an omnichannel retailing investment their top priority for the next three to five years (Fibre2fashion 2022).

The retail industry report revealed a higher level of omnichannel purchase behavior in all retail ranges (Deloitte 2020). For instance, the Saigon Co-op, a supermarket operator, reported ten times more telephone orders and the number of visits to its website had risen ten times since January 2020. Fifteen years ago, the average customer used two channels during their search and purchase; however, the number of channels increased to six by 2016 (Chahal 2016). According to McKinsey and Company's (2021) retail report, customers expect more online purchases, and e-commerce is projected to reach 40% of sales post-pandemic.

The sampling method is cluster purposive sampling for the three most prominent cities in Vietnam: Hanoi, Da Nang, and Ho Chi Minh. The three cities have similar demographic characteristics to the total population and represent three economic hubs of North, Central, and South Vietnam.

The final dataset for fsQCA analysis contains 390 cases out of 489 participants (79.8%) who completed the survey. The number of participants is sufficient for this study as QCA can test a model with a few cases to extensive sample data (Berg-Schlosser et al. 2012; Greckhamer et al. 2013). Approximately 27.7% of the participants are male, and 72.3% are female. The participants comprised shoppers who were 20 and under (21.5%), aged 21–40 (62.1%), aged 41–54 (5.1%), and 50 and above (1.3%). Overall, the participants are female millennial omnichannel shoppers.

### Measurement

The survey instrument consists of 18 questions from the constructs included in this study. The participants answered the questions using a five-point scale (1—strongly disagree, 2—disagree, 3—neutral, 4—agree, and 5—strongly agree). Appendix A shows the survey questionnaire.

### Reliability and validity measures

We used Cronbach Alpha to test the data's internal consistency and reliability (Saunders, Lewis, and Thornhill, 2019). The coefficient values from 0.6 and above indicate that the questions in the scale consistently measure the same constructs and, therefore, higher reliability (Hundleby and Nunnally 1968). Furthermore, all constructs used in this study have a Cronbach alpha of 0.799, higher than 0.6, proving that internal consistency exists. The Cronbach alpha of each variable group also reports satisfactory results of 0.65 for personal factors, 0.757 for environmental factors, and 0.674 for behavioural factors.

Statistically, there are several methods to use for normality testing. Among them are skewness and kurtosis tests. For a typical data distribution, the skewness value should be near zero. Hair et al. (2019) stated that the acceptable range is from 1 to + 1 to avoid normality problems. Other researchers suggested that skewness and kurtosis values between − 2 to + 2 would not indicate any concern for normality (Gravetter and Wallnau 2010). The results from the test showed that the data collected has a skewness value and kurtosis value of less than 2, which indicated that there is no significant deviance from a normal distribution for the collected data. In addition, the predicted normality P–P plot for the regression residuals was used to make a further normality assessment from the residual analysis. The standardised projected value formed a line with the standardised residuals indicating an adequate level of normality.

The dataset was also tested for construct validity using convergent and discriminant validity. Convergent validity describes the relationship between responses from various variables when assessing the same construct (Hair et al. 2019). The test is based on the average variance extracted (AVE) with the rule of thumb that the AVE must be greater than or equal to 0.5 to prove its validity (Hair et al. 2019). Table 2 shows the environmental factors (EF) having an AVE value of 0.53. In contrast, personal factors (PF) and behavioural factors (BF) have an AVE value between 0.3 and 0.5 (attitude of 0.34 and perceived behavioural control of 0.36). Although the last two factors did not meet the recommended AVE value of 0.5 (Fornell and Larcker 1981), the composite reliability (CR) values of all factors are higher t,han 0.6 which signifies a reasonable degree of internal reliability (Lam 2012).

**Table 2:** Squared inter-construct correlations

					1	2	3
		CR	AVE	MSV	EF	PF	BF
1	Environmental factors (EF)	0.757	0.532	0.114	0.730		
2	Personal factors (PF)	0.65	0.339	0.270	0.337	0.582	
3	Behavioural factors (BF)	0.674	0.362	0.270	0.232	0.520	0.602

Discriminant validity proves that the measured variables of constructs are distinct and not highly correlated (Hair et al. 2019). This means that the average shared variance (ASV) and maximum shared variance (MSV) should be less than the average variance extracted (AVE) (Rajalahti and Kvalheim 2011). Table 3 demonstrates the discriminant validity where the inter-construct correlations do not exceed 0.85 and the AVE results of all factors are higher than the MSV values, thus indicating good discriminant validity.

**Table 3:**
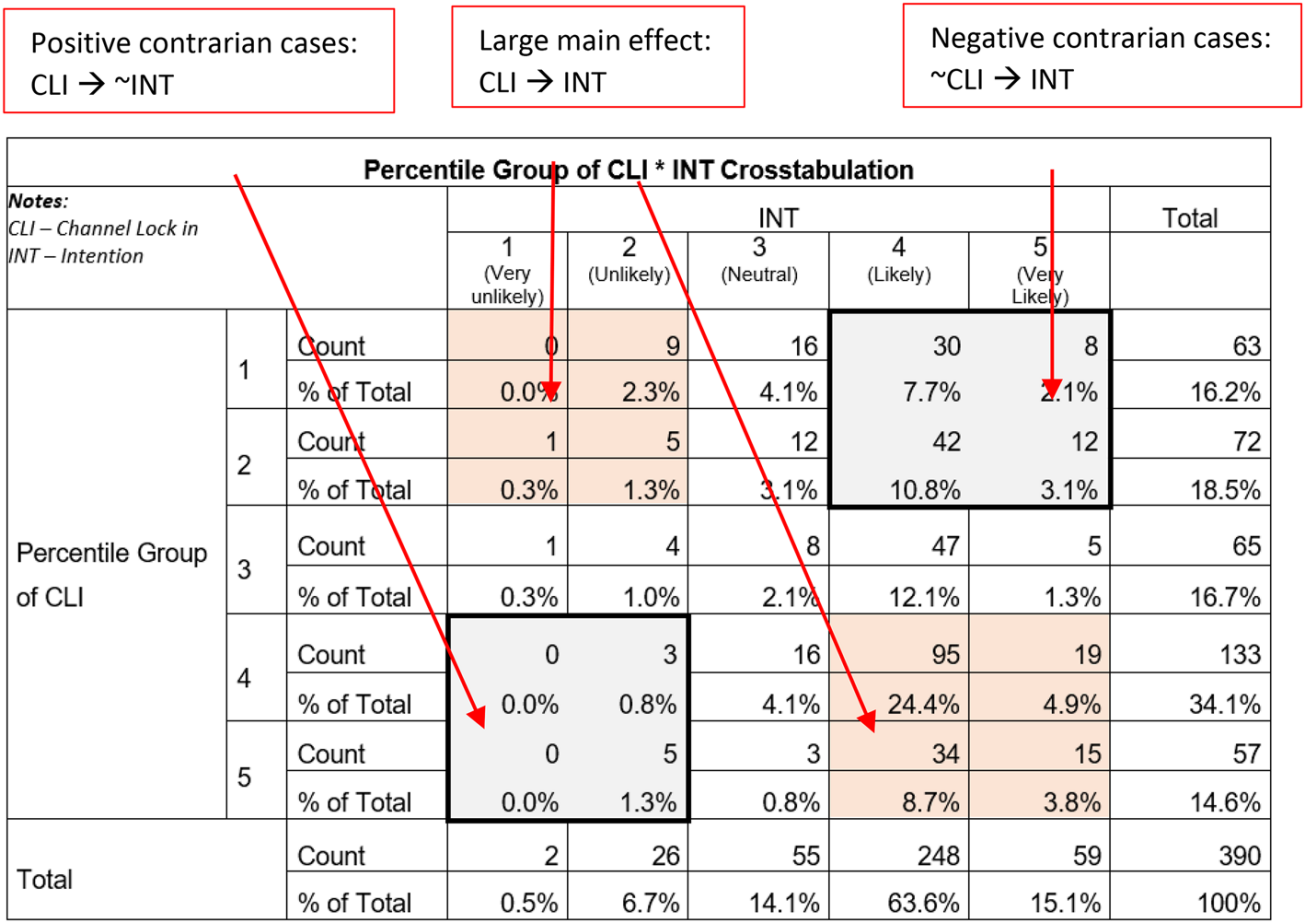
Percentile Group of Channel Lock in * Intention Crosstabulation

### Contrarian case analysis

Contrarian cases exist for cases "having low X with high Y and high X with low Y occur even when the relationship between X and Y is positive, and the effect size of the relationship is large" (Woodside 2014, p.1). Moreover, Pappas and Woodside (2021) expected the existence of cases that are not explained by the main effects and are most likely excluded in a typical variance-based approach. Hence, we performed a contrarian case analysis to determine if such cases were within our sample.

To prepare for the analysis, we ensured that all indicators in the questionnaire were positively worded so that we did not have to do reverse coding. Moreover, we did not include cases with missing values in the sample. We treated each construct as a “quasi-interval” because the indicators measure similar things. For example, the latent values of CCS (or ATD_5_) and OPB (or SN_1_) are the same, as both contain only one indicator. Next, we obtained the mean score (av) for each construct having multiple indicators (*n*), namely CLI, ADM, PSO, PSE, and PFC, using formulas (3)–(7), as follows:

3 $$CLI_{av} = \frac{{\mathop \sum \nolimits_{i = 1}^{n} ATD_{i} }}{n},\;where\;n = 4$$

4 $$ADM_{av} = \frac{{\mathop \sum \nolimits_{i = 6}^{9} ATD_{i} }}{n},\;where\;n = 4$$

5 $$PSO_{av} = \frac{{\mathop \sum \nolimits_{i = 2}^{4} SN_{i} }}{n},\;where\;n = 3$$

6 $$PSE_{av} = \frac{{\mathop \sum \nolimits_{i = 1}^{n} PBC_{i} }}{n},\;where\;n = 2$$

7 $$PFC_{av} = \frac{{\mathop \sum \nolimits_{i = 3}^{4} PBC_{i} }}{n},\;where\;n = 2$$

To determine contrarian cases, we then ranked the cases and used the quintiles to divide the sample into five (5) equal groups. After that, we used descriptive statistics using Crosstabs to get the count and total percentages of cases. Tables 3 and 4 are sample 5 × 5 matrices showing the variables’ association and outcome. Appendix B presents other matrices.

**Table 4:**
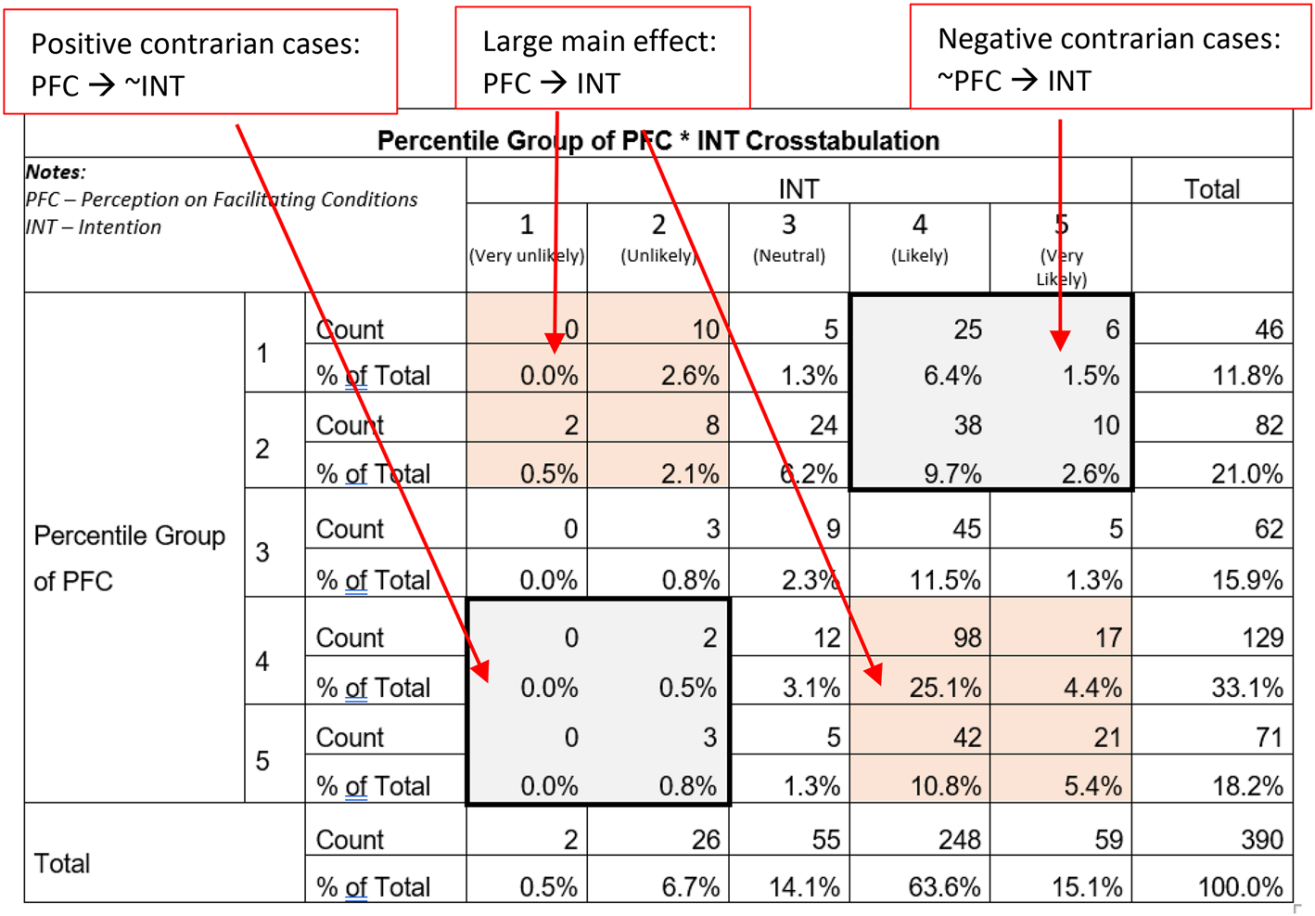
Percentile Group of Perception on Facilitating Conditions * Intention Crosstabulation

We discovered that there are relationships among the variables other than the main effects, indicating the presence of contrarian cases. For instance, a high degree of CLI leads to an increased likelihood of switching channels. However, in some cases, a low degree of CLI still leads to an increased likelihood of switching channels (refer to the section at the bottom left and top right of Table 3). This occurrence is similar for PFC, as illustrated in Table 4, and for the rest of the constructs. These findings show variations in the conditions that result in the same positive (or negative outcome). Therefore, a configurational analysis must be performed to include these potentially significant cases in the study.

### fsQCA analysis, findings, and discussions

**Fig. 3:**
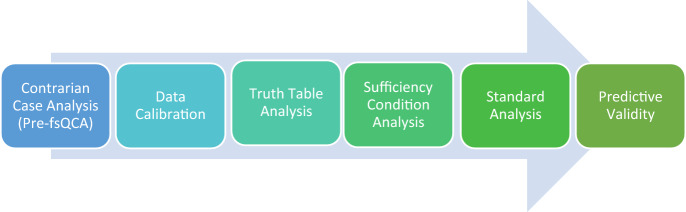
fsQCA workflow

The complete fsQCA workflow presents in Fig. 3. We used the steps outlined in the study of Pappas and Woodside (2021) for our fsQCA analysis. The Contrarian Case Analysis, which is the first step in the workflow, is presented in the previous section of this paper.

#### Data calibration

Calibration is the first step in fsQCA. First, we transformed the Likert metrics into fuzzy values with varying degrees of membership (Mekawy and Elbaz 2020). Next, we converted the ordinal data on the 5-point Likert scale into varying membership scores between 0 and 1 using the fsQCA 3.0 software that was developed by Ragin and Davey (2016). Finally, the fsQCA software transforms all observed variables to membership scores using a logarithmic function (Dul, 2016).

Greckhamer et al. (2018, p. 7) stated that effective calibration is a “half-conceptual, half-empirical process of identifying thresholds”. Some studies create calibrated set of membership according to the theoretical and collected knowledge of the cases (De Villiers, 2017). This study mainly uses the direct calibration method to transform the variables into sets. Since the data are skewed, we used the calibrate function of the fsQCA software and the following thresholds: 0.95 for full membership, 0.5 for crossover point of membership ambiguity (fuzziness), and 0.05 for full non-membership as suggested by Pappas et al. (2016). The full membership corresponds to a score of 4 and above. The crossover point corresponds to a score of 3. The full non-membership corresponds to a score of 1 and below. We show a summary of the data calibration for the primary variables in Table 5.

**Table 5:** Data Calibration Measures

Type	Variable name	Abbreviations	Likert scale	Crisp/fuzzy set scale
Antecedent condition	Age	Age	Demographic info on survey data	*Crisp set* Full non-membership: 0 = 40 and below (millennials) Full membership 1 = 41 and above
Antecedent condition	Gender	Gender	Demographic info on survey data	*Crisp set* Full non-membership: 0 = Female Full membership: 1 = Male
Antecedent conditions	Channel lock-in	CLI	5-point Likert scale 1 = Strongly disagree 2 = Disagree 3 = Neutral 4 = Agree 5 = Strongly agree	*Fuzzy set* Full membership: 0.95 = 4 and above Cross-over point: 0.5 = 3 Full non-membership: 0.05 = 1 and below
	Cross channel synergy	CCS		
	Attribute-based decision making	ADM		
	Other past switching behaviour	OPB		
	Pressure to switch from others	PSO		
	Perceived self-efficacy	PSE		
	Perception of facilitating conditions	PFC		
Outcome	Channel switching intention	INT		

Table 6 shows configurational data of the calibrated fuzzy sets of the causal conditions (CLI, CCS, ADM, OPB, PSO, PSE, and PFC) and the fuzzy score of the INT outcome.

**Table 6:** Calibrated Fuzzy sets

CLIc	CCSc	ABMc	OPBc	PSOc	PSEc	PFCc	INTc
0.41	0.95	0.95	1	0.98	0.32	0.32	1
0.5	0.95	0.82	0.95	0.88	0.95	0.5	0.5
0.82	0.95	0.82	1	0.95	0.82	0.82	0.95
0.5	0.95	0.95	0.5	0.88	0.82	0.5	0.95
0.82	0.95	0.82	0.95	0.88	0.5	0.5	0.5
0.68	0.5	0.9	0.05	0.05	0.99	0.5	1
0.68	0.95	0.95	0.95	0.73	0.82	0.95	1
1	1	1	1	1	0.95	0.5	0.18
0.82	0.95	0.95	0.95	0.95	0.95	0.82	0.95
1	1	1	0.95	0.95	0.95	0.95	0.95

#### Truth table analysis

QCA allows researchers to compare cases to determine patterns of causal conditions that lead to the outcome and will enable them to analyse complex interactions between conditions. In doing so, we built a truth table and provided all logical combinations of causal conditions. We did not examine the conditions in isolation but treated them as components of configurations (De Villiers, 2017).

The truth tables for the INT and ~ INT outcomes are generated with $2^{k}$ rows, where 2 represents the presence or absence conditions, and k = 7 represents the number of causal conditions, totalling 128 combinations. Table 7 shows snapshots of the truth tables based on the number of cases found in the calibrated data.

**Table 7:**
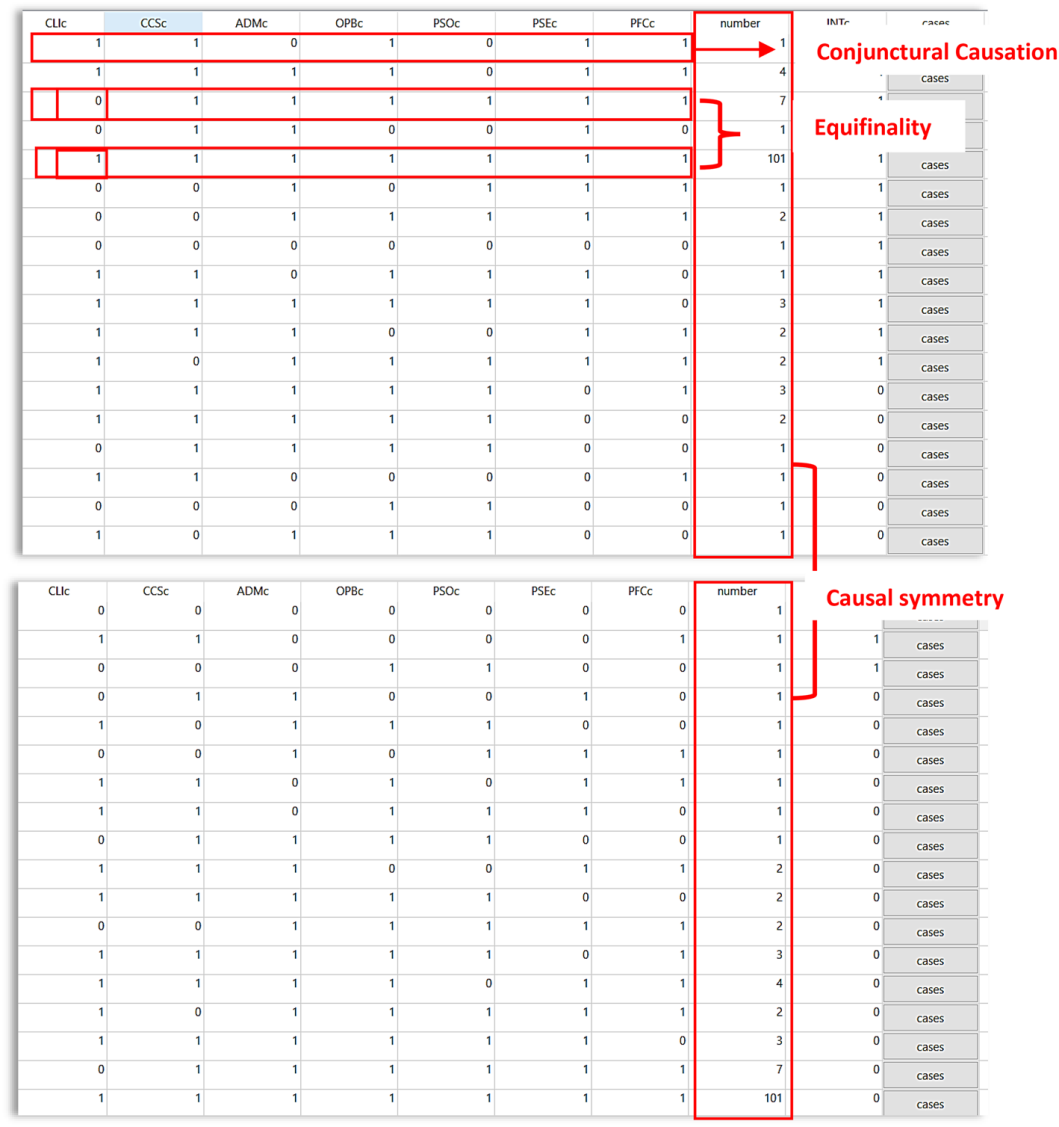
Cases found in the calibrated data

The truth tables account for how QCA supports the principles of causal complexity, which are conjunctural causation, equifinality, and causal asymmetry (Misangyi et al. 2017). First, conjunctural causation is exhibited in the first truth table (Table 7). The table shows seven (7) causal conditions that are in conjunction. For instance, the first row of the first truth table contains the following conditions: CLI, CCS, ~ ADM, OPB, ~ PSO, PSE, and PFC, which can lead to the presence of the intention outcome. It shows a combination of causal conditions instead of the independent net effect of each condition, which relates to the outcome. QCA supposes that the outcomes are caused by (multiple) individuals or combinations of causal conditions rather than a single outcome from several conditions (Mekawy and Elbaz 2020; Sukhov et al. 2021).

Second, equifinality occurs when the same outcome results in a particular condition(s) presence or absence. There will be multiple paths or solutions that can lead to the same outcome. For instance, the 5th row of the first truth table shows that the presence of CLI, CCS, ADM, OPB, PSO, PSE, and PFC conditions will produce a presence of an intention to switch channels. Evidently, given the same conditions but without the CLI condition (as shown in the 3rd row of the first table), the intention to switch channels still exists.

Third, causal asymmetry is assumed when the presence and absence of the outcome require different explanations (Berg-Schlosser et al. 2012). Finally, QCA compares cases and examines the influence of configurations of causal conditions on the outcome (Leppänen et al. 2019). Specifically, it tests the presence and absence of causal conditions that regulate the presence or absence of the outcome (Sukhov et al. 2021). We used two (2) truth tables to identify the different configurations that will explain the presence and absence of the INT outcome. Each truth table contains varying configurations for the outcomes of interest.

#### Sufficiency condition analysis

QCA also allows researchers to analyse the individual and combined necessity and sufficiency of the causal conditions relative to the outcome (Sukhov et al. 2021). Necessity means that condition(s) must be present in all cases for the presence or absence of the outcome. In contrast, sufficiency implies that a condition(s) is enough for the presence or absence of the outcome (Leppänen et al. 2019). In addition, sufficient conditions must meet certain conditions for the outcome (Rihoux and Ragin, 2009).

Rihoux and Ragin (2009) describe consistency as the extent to which the empirical evidence or causal combination leads to the set-theoretic related question or outcome. Greckhamer et al. (2018) point out that consistency can assess the model’s validity, meaning a low consistency score suggests poor explanatory power for the outcome. On the other hand, Rihoux (2006) explains that coverage indicates the relevancy or importance of a configuration. De Villiers (2017) provides the formula on how to compute the consistency and coverage for each causal combination using formulas 8 and 9, where $X_{i}$ is the membership score in the causal combination and $Y_{i}$ is the membership score in the outcome, and “*min*” indicates the lower value between *X*_*i*_ and *Y*_*i*_.

8 $${\text{Consistency}} \left( {X_{i} \le Y_{i} } \right) = \frac{{\sum \left( {\min \left( {X_{i} ,Y_{i} } \right)} \right)}}{{\sum \left( {X_{i} } \right)}}$$

9 $${\text{Coverage}} \left( {X_{i} \le Y_{i} } \right) = \frac{{\sum \left( {\min \left( {X_{i} ,Y_{i} } \right)} \right)}}{{\sum \left( {Y_{i} } \right)}}$$

We performed a sufficiency test for this study using consistency and coverage scores. According to Elliot (2013), the data should drive the exact cut-off point for the consistency score. Pappas et al. (2018) recommend having a consistency threshold of 0.85 and above for a configuration to be sufficient. The threshold is slightly higher than the minimum recommended value of 0.75 (De Villiers 2017). For management research, a consistency threshold of 0.80, the default value in the fsQCA software, is acceptable (Ragin 2018). Thus, we use consistency thresholds of at least 0.8 for the INT and ~ INT outcomes. We also set the frequency threshold to 2 since we used 135 samples after performing data calibration (Fiss 2011; Ragin 2008). On the other hand, we ensured that the coverage score for each sufficient configuration was within the score range of 0.2–0.6, as suggested by De Villiers (2017). We considered the suggestion of Elliot (2013), who pointed out that high consistency will cause lower coverage because this is a typical trade-off between consistency and coverage. In addition, we examined the Proportional Reduction in Inconsistency (PRI) scores to confirm if they are close to the raw consistency scores (Pappas and Woodside 2021).

After setting a frequency cut-off of 2 and a consistency threshold of 0.8, the new truth tables (Table 8) for the INT and ~ INT outcomes produced nine (9) logical combinations covering 126 cases. The table shows high raw consistency scores of more than 0.94. The cases that do not meet all the criteria were removed since these cases are not significant to the outcome and are likely to produce false positives (Dul 2016). Thus, the remaining cases were used for further analysis and expected to contain sufficient configurations for the outcomes of interest.

**Table 8:** Sorted truth tables after setting the frequency and consistency thresholds

OPBc	PSOc	PSEc	PFCc	Number	INTc	Cases	Raw consist	PRI consist	SYM consist
INT outcome									
1	0	1	1	4	1	Cases	0.973967	0.952335	0.95603
1	1	1	1	7	1	Cases	0.973503	0.96093	0.96093
1	1	1	1	101	1	Cases	0.972785	0.966485	0.974508
1	1	1	1	2	1	Cases	0.972039	0.942342	0.942342
1	1	1	0	3	1	Cases	0.960362	0.935045	0.935045
0	0	1	1	2	1	Cases	0.955799	0.894737	0.911995
1	1	1	1	2	1	Cases	0.95394	0.919606	0.919606
1	1	0	1	3	1	Cases	0.94726	0.902825	0.914166
1	1	0	0	2	1	Cases	0.945555	0.887169	0.887169

OPBc	PSOc	PSEc	PFCc	Number	~ INTc	Cases	Raw consist	PRI consist	SYM consist
~ INT outcome									
0	0	1	1	2	0	Cases	0.61634	0.0863394	0.0880048
1	1	0	0	2	0	Cases	0.571911	0.112831	0.112831
1	1	1	1	2	0	Cases	0.54301	0.0576582	0.0576582
1	1	0	1	3	0	Cases	0.503273	0.0847692	0.085834
1	0	1	1	4	0	Cases	0.477748	0.0438	0.0439699
1	1	1	1	2	0	Cases	0.473132	0.0803935	0.0803936
1	1	1	0	3	0	Cases	0.429406	0.0649546	0.0649547
1	1	1	1	7	0	Cases	0.348297	0.0390699	0.0390699
1	1	1	1	101	0	Cases	0.208506	0.0252819	0.0254917

For the truth table of the INT outcome, the PRI consistency scores of each combination are high and close to the raw consistency scores. However, for the negation of the outcome, some PRI consistency scores are weak (below 0.5), indicating significant inconsistencies (Greckhamer et al. 2018). It is safe to conclude that the configurations are not sufficient for the ~ INT outcome (as confirmed in Table 10).

#### Standard analysis and discussions

The fsQCA 3.0 software uses the Quine-McCluskey algorithm for performing the Boolean minimisation. The software produces different solution types (see Appendix C), namely, intermediate (QCA-IS), parsimonious (QCA-PS), and complex (QCA-CS), to produce the causal configurations.

QCA-CS does not use data minimisation techniques to simplify the solution (Schneider and Wagemann 2012). Thus, the solution produced is highly complex and makes the interpretation of findings difficult. The QCA-PS generates the most parsimonious solution. Baumgartner and Thiem (2017) used QCA-PS to identify causal relationships with strong empirical evidence. However, it excludes significant conditions because it uses minimisation techniques to produce a more straightforward solution (Dușa 2019). The QCA-IS solution is considered the best hybrid solution to identify an underlying causal structure (Dușa 2019). Thiem (2019) highlighted some issues with QCA-CS and QCA-IS because artificial data are added to the empirical data that can draw incorrect inferences. Nonetheless, our study will present the intermediate solution to provide the findings.

Table 9 shows the intermediate solution of the INT outcome with an overall solution coverage of 0.95 and solution consistency of 0.74. It contains three (3) configurations with corresponding consistency and coverage scores. According to Ragin (2008), a configuration should have a raw coverage score of 0.2 to confirm its sufficiency. Since configuration 3a failed to meet the coverage threshold, we did not consider it to be a sufficient configuration for the presence of the INT outcome to occur. Therefore, only configurations 1a and 2a were sufficient for the INT outcome, given their high consistency and coverage scores of at least 0.96 and 0.61, respectively (numbers presented in bold - Table 9).

**Table 9:** QCA-IS for the INT outcome

Configuration	CLI	CCS	ADM	OPB	PSO	PSE	PFC	Raw coverage	Unique coverage	Consistency
1a	●	●	●	●	●			**0.617137**	0.0517926	**0.962866**
2a			●	●	●	●	●	**0.657825**	0.0924801	**0.960899**
3a	●	●	●		o	●	●	0.182605	0.0242292	0.96711
Frequency cutoff: 2 Consistency cutoff: 0.945555				Solution coverage: 0.733847 Solution consistency: 0.951791						

(●) denotes a presence of a condition; (o) denotes an absence/negation of a condition; Blank space denotes a "Don’t care" condition

*Configuration 1a* shows a combination of CLI, CCS, ADM, OPB, and PSO conditions among the participants' behaviour that led to channel switching intention.

The configuration, which reveals that the participants exhibit behaviours relating to personal and environmental factors (except behavioural factors), shows the presence of an intention to switch channels. The participants perceived that one channel could be both search and purchase attractive, different channels can be search or channel attractive, and different channels can excel in search or purchase attributes. They are also influenced by other people's switching behaviour (social norms) and pressured to switch from others (descriptive norms). Both personal and environmental factors have a significant influence on customers to switch channels.

Past research found that factors such as customers’ perception of search and purchase attractiveness, benefits of switching, attributed-based decision-making, and self-efficacy, can individually influence webrooming or showrooming intention (Arora et al. 2017; Flavián, Gurrea, and Orús 2020; Kang 2019). The participants' behaviour confirms the research shopper model findings that describe how low channel lock-in and cross-channel synergy are the two main reasons for channel switching without the need for significant evidence for attributed-based decision-making (Verhoef et al. 2007). Also, Verhoef et al. (2007) and Nguyen et al. (2022) describe how differing perceptions of channels' search and purchase attributes of customers lead to a channel-switching intention. Since the participants are millennial shoppers, the participants' behaviour reinforces the study of Maggioni et al. (2020) which describes how a significant percentage of young customers actively seek information and compare one website to another.

According to Pavlou and Fygenson (2006), a person's circle of influence impacts the customer's perception of whether channel-switching behaviour is accepted, encouraged, or applied. Nguyen et al. (2022) report that customers are induced to conform to social norms and trust reference sources. However, no research studies have reported that others' past behaviours and pressure to switch (or subjective norms) can independently influence channel-switching intention. Chou et al. (2016) suggested that subjective norms and past switching behaviour must combine with attitude toward switching, switching cost, and variety-seeking for a sufficient configuration that contributes to a cross-channel free-riding intention. Likewise, Arora et al. (2020, 2021) stressed the combination of perceived switching value and customer self-efficacy or perceived behavioural control on webrooming and showrooming intention.

*Configuration 2a* shows a combination of the presence of ADM, OPB, PSO, PSE, and PFC conditions among the participants' behaviour led to a presence channel switching intention.

The configuration, which reveals that participants exhibit behaviours relating to personal, environmental, and behavioural factors, shows the presence of an intention to switch channels. The participants perceive that different channels can excel in search or purchase attributes. Thus, they will switch between online and offline channels when they perceive that one channel is better. This behaviour stems from customers' need for comparative evaluations before purchasing decisions (Maggioni et al. 2020). Likewise, they will switch when they see other people's switching behaviour and outcome or when under the influence of other people, such as friends and families, salespersons, and social influencers. Moreover, they tend to believe that they have control over the switching action and outcome and have the ability, skills, and conditions (i.e. time, resource, technology, location) to perform the switching task.

The intermediate solution of the ~ INT outcome (Table 10), which shows three (3) potential configurations to explain the absence of the intention to switch channels, has an overall solution consistency score of 0.19 and a coverage score of 0.77. Since none of the configurations met the consistency threshold, they are not considered sufficient for the ~ INT outcome.

**Table 10:** QCA-IS for the ~ INT outcome

Configuration	CLI	CCS	ADM	OPB	PSO	PSE	PFC	Raw coverage	Unique coverage	Consistency
1c	●	●	●	●	●			0.673637	0.461027	0.202949
2c			●	●	●	●	●	0.687421	0.598858	0.193895
3c	●	●	●		o	●	●	0.446927	0.3326698	0.457064
Frequency cutoff: 2 Consistency cutoff: 1				Solution coverage: 0.766793 Solution consistency: 0.19204						

(●) denotes a presence of a condition; (**o**) denotes an absence/negation of a condition; Blank space denotes a "Don’t care" condition

#### Predictive validity

According to Woodside (2014), solutions (or models) should be tested for predictive validity to demonstrate how well the model predicts the outcome using different or additional samples. We accomplished this by following the process suggested by Pappas and Woodside (2021). First, we randomly divided the sample into two (2) parts: the subsample and the holdout sample. Second, we run fsQCA analysis for the subsample to produce the solutions, as shown in Table 11. The table shows two (2) configurations of interest: Configuration 1d and Configuration 6d. These configurations are similar to those found in QCA-IS for the INT outcome. Refer to Appendix D for the fsQCA solutions of the subsample and holdout sample.

**Table 11:** Solution from the Subsample

Configuration	CLI	CCS	ADM	OPB	PSO	PSE	PFC	Raw coverage	Unique coverage	Consistency
1d	●	●	●	●	●	●		0.622293	0.0269969	0.974915
2d	●	●	●	●	●		●	0.604792	0.0094955	0.971682
3d	●	●	●	●		●	●	0.609012	0.0137156	0.975447
Frequency cutoff: 2 Consistency cutoff: 0.958946				Solution coverage: 0.645504 Solution consistency: 0.969158						

(●) denotes a presence of a condition; (o) denotes an absence/negation of a condition; Blank space denotes a “Don’t care” condition

**Fig. 4:**
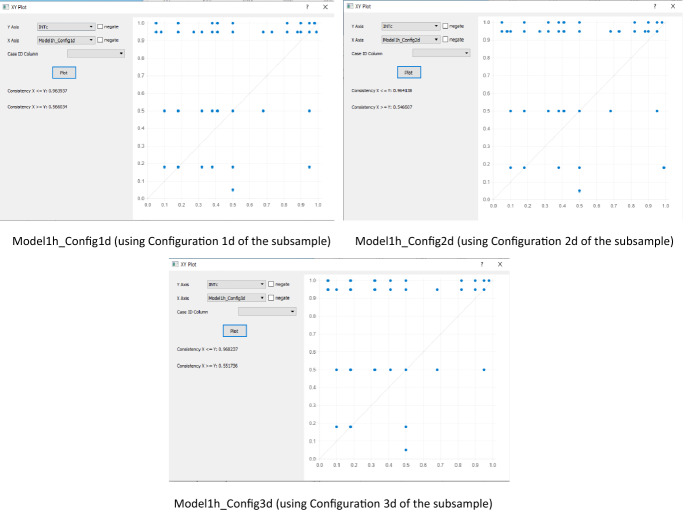
XY-plot for the models in the Subsample using the Holdout sample

After we obtained the configurations in the subsample, Configuration 1d, 2d, and 3d were transformed into models using the *fuzzyand *(*CLI*, *CCS*, *ADM*, *OPB*, *PSO*, *PSE*), *fuzzyand *(*CLI*, *CCS*, *ADM*, *OPB*, *PSO*, *PFC*), and *fuzzyand *(*CLI*, *CCS*, *ADM*, *OPB*, *PSE*, *PFC*)*, respectively*. After that, we plotted the new models against the INT outcome of the holdout sample to test the predictive power (Fig. 4).

The XY-plot (shown on the left in Fig. 4) produced a set-theoretic consistency score of 0.96 and coverage score of 0.57 for Model1h_Config1d, consistency score of 0.96 and coverage score of 0.575for Model1h_Config2d, and consistency score of 0.97 and coverage score of 0.55 for Model1h_Config3d. The high consistency and coverage scores show that the models are subsets of the main model (full sample) for the INT outcome.

Table 12 compares the configurations obtained from the three (3) samples used in this study. We observed that the configurations are primarily consistent among the samples. For example, the subsample and holdout samples contain a similar configuration (3d and 1e) nearly identical to the full sample (1a). We also observed that all models achieved a relatively high consistency score of over 0.9, which is considered valuable for theory advancement.

**Table 12:** Comparative analysis of the samples

Configuration No	Sufficient configurations	Raw coverage	Consistency
*Full sample*			
1a	CLI●CCS●ADM●OPB●PSO	0.617137	0.962866
2a	ADM●OPB●PSO●PSE●PFC	0.657825	0.960899
*Subsample*			
1d	CLI●CCS●ADM●OPB●PSO●PSE	0.622293	0.974915
2d	CLI●CCS●ADM●OPB●PSO●PFC	0.604792	0.971682
3d	CLI●CCS●ADM●OPB●PSE●PFC	0.609012	0.975447
*Holdout sample*			
1e	ADM●OPB●PSO●PSE●PFC	0.590529	0.960173
1e	CLI●CCS●ADM●OPB●PSE●PFC	0.636864	0.964106

## Implications and future work

### Theoretical and methodological implications

Most published studies in omnichannel switching literature use symmetric approaches. This study challenges theoretical underpinnings by demonstrating that omnichannel channel-switching behaviours can be explained from a configurational perspective. For this study, we use an asymmetric approach to understand the behavioural complexity of omnichannel shoppers. We use QCA not to build a model that predicts high and low scores but models that produce sufficient configurations for the presence and absence of the channel switching intention outcomes. We also included the positive and negative contrarian cases in our analysis, which are disregarded in most symmetric studies (Wu et al. 2016). We presented the intermediate solution to provide sufficient configuration for the presence of the channel switching intention. However, no sufficient configuration was found regarding the absence of the channel switching intention.

An important contribution of this research is the step-by-step process of data analysis in fsQCA. It provides a detailed account of all the pre-, during, and post-QCA processes for transparency and replicability. Like most QCA researchers, we argue that this paper can pave the way to establish configuration effects that are not yet present in the existing channel switching literature and are very unlikely to be produced in conventional quantitative method-based studies (Paykani et al. 2018). Soda and Furnari (2012) also contended that the plausibility of the configurations could be treated in the same way that architects and engineers envision their future work.

This study provide new configurational perspectives that can help researchers and scholars explain the complexity of channel-switching behaviour and deepen their understanding of customer behaviour. Accordingly, this study can help them assess the relevance of certain antecedent conditions in a specific omnichannel context. It also highlights the differences in channel-switching behaviours of customers during their search and purchase journey. This study shows the combinations of causal conditions on why customers switch channels and the configurations on why they do not. These perspectives help identify strengths and pain points in the customer experience. Therefore, we claim to be one of the pioneering QCA studies in this field.

### Practical and managerial implications

In terms of practical contribution, this study reveals the diverse nature of the channel-switching behaviour of omnichannel customers, which retailers can use to support their business and marketing strategies. Retailers should deeply understand the different factors underlying channel-switching behaviours and consider their approach to handling these customer behaviours (Maggioni et al. 2020). Doing so helps them identify pain points and critical moments in the customer journey that lead customers to have the intention to switch channels. It also presents an opportunity for them to prevent the customers from switching channels and maximise the different stages of the customer journey.

First, the configurations included personal factors such as channel lock-in, cross-channel strategy, and attribute-based decision-making that contribute to the presence of an intention to switch channels. Retailers should examine crucial channel attributes that will attract and support customers during their search and purchase journey and incentivise them to use online and offline channels. In doing so, retailers should consider online search channel attributes such as time-saving, information-rich, and ease of use, as well as offline search channel attributes such as readiness, in-person shopping enjoyment, and “touch and feel” features (Nguyen et al. 2022). Since channel switching is also influenced by channel diversification (Kokho Sit, Hoang, and Inversini, 2018) and the utilisation of mobile devices for search and purchase (Wang, Malthouse, and Krishnamurthi, 2015), retailers should explore the effect of mobile devices to customers' channel-switching behaviours. The use of smartphones has led to m-commerce growth that generated “mobile showrooming” behaviours (Viejo-Fernández, Sanzo-Pérez, and Vázquez-Casielles, 2020).

Moreover, Crawford (2005) supposed that shoppers follow a complex purchase journey using different channels to achieve their purchase goals. Nguyen et al. (2022) affirmed that customers are switching simultaneously between offline and online channels, and the customer journey has gone beyond conventional showrooming and webrooming. This makes it hard for retailers to identify channel-switching causes. Grant et al. (2010) suggested that retailers gather clickstream data of customers to know their browsing behaviour, offer personalisation, and improve purchase engagement. This was also supported by Frishammar et al. (2018), who suggested that retailers embrace digitisation.

Second, the configurations included environmental factors such as others' past switching behaviour and pressure to switch from others that contribute to the presence of an intention to switch channels to occur. For millennial customers, influencers play a significant role in their customer journey. Hence, retailers must adopt influencer marketing using (electronic) word-of-mouth techniques and key opinion leaders as they are trustworthy sources among Gen Zs and millennials.

Third, the configurations included behavioural factors such as perceived self-efficiency and perceived facilitating conditions that contribute to the presence of an intention to switch channels. The switching behaviour in the omnichannel context often involves a higher level of technical efficiency, knowledge, and resources. This means retailers must invest more in online or offline platforms to ensure customers’ easy and convenient purchase journeys. The conditions also inform the consumer journey design of online customers with predictable switching behaviour. For products with high-involvement levels, channel switching will likely happen as the customers spend more time and resources on decision-making. Verhoef et al. (2007) also referred to product involvement as one of the determinants of switching behaviour related to the degree of in-store value-taking. Thus, the journey design of customers with high-involvement purchases will be more complex involving multiple touchpoints (Daunt and Harris, 2017).

### Limitations and future direction

We also acknowledged the limitation of the QCA software used in this study. Although it can provide objective configurations, it lacks the flexibility of algorithmic choices. The software uses the popular Quine-McCluskey algorithm to process the Boolean functions. Although it is suitable for processing moderately complex Boolean functions, it has a memory restriction that can only process approximately 12 condition factors (Duşa and Thiem 2015). Since our study used only seven (7) constructs, the software somehow served our purpose and provided the solutions required to explain the phenomenon under study. We suggest that future researchers use fewer than eight constructs because it is unnecessary to consider all influencing factors and difficulty in interpreting the solutions. Likewise, this study captured fewer cases after calibration. Although they are more than enough (Thiem 2022), we recommend that future researchers retain as many cases as possible, preferably 80% (Greckhamer et al. 2018).

As a future direction, we suggest that researchers consider other causal conditions influencing channel switching among omnichannel customers, such as anxiety, interaction need, and privacy (Cai and Lo 2020); and situational factors such as shopping convenience, experiential attributes, switching cost, and consistency of the shopping experience across all channels (Pookulangara et al. 2011). This study also suggests extending the generalizability of the findings by employing more (representative) sample groups of omnichannel customers. Future research can analyse other customer groups, not only electronic goods customers.

Because of the limited literature on using QCA for channel switching in an omnichannel context, this paper primarily utilised the existing theoretical and empirical knowledge from symmetric-based studies to explain the causal configurations. Therefore, most of the "whys" in these configurations are general explanations from these studies. Hence, they may only partially explain the complex channel-switching phenomenon. We suggest that future researchers find QCA studies to support their findings.

## Conclusion

In this paper, we explained the nuances of a complex phenomenon of customers' switching behaviour in an omnichannel retail context. We used a novel methodological approach to model multiple behavioural realities. The research examines the interplay among personal factors such as channel lock-in, cross-channel synergy, and attribute-based decision making (ADM); environmental factors such as others’ past switching behaviour (OPB), pressure to switch from others (PSO); and behavioural factors such as perceived self efficacy (PSE) and perception on facilitating conditions. These factors are antecedents of customers' channel-switching intention in an omnichannel context.

This paper provided analyses of sufficiency to reveal significant causal configurations that explain the presence and absence of intention to switch channels. These configurations achieve high set-theoretic consistency and coverage scores. The result of the analysis indicated two (2) sufficient configurations produced by the QCA-IS solution that led to a presence of an intention to switch channels. The first configuration offers a combination of CLI, CCS, ADM, OPB, and PSO conditions, and the second configuration offers a combination of ADM, OPB, PSO, PSE, and PFC conditions. Both configurations contain ADM, OPB, and PSO conditions that highlight the importance of personal factors and environmental factors needed for the presence of an intention to switch channels. However, no sufficient configurations were obtained that indicate an absence of intention to switch channels. Furthermore, we implemented predictive validity to confirm the model’s predictive performance. The configurations obtained from the subsample and holdout samples achieved high theoretic consistency and coverage scores indicating good predictive power. Therefore, we conclude that this study fills the research gap and enables the advancement of theory, method, and practice of channel switching in omnichannel retailing.

## Appendix A: Questionnaire

**Table:**

Construct	Items	5-point Likert scale
Attitude towards channel switching (ATD)/personal factors	*ATD 1* (CLI1)—the trustworthiness of online channel for search *ATD 2* (CLI2)—the trustworthiness of offline channel for search *ATD3* (CLI3)—the risk of online channel for purchase *ATD4* (CLI4)—the risk of offline channel for purchase *ATD5* (CCS)—level of benefits you get from switching channels during search and purchase *ATD6* (ADM1)—search attractiveness of online channels *ATD7* (ADM2)—search attractiveness of offline channels *ATD8* (ADM3)—purchase attractiveness of online channels *ATD9* (ADM4)—purchase attractiveness of offline channels	1—Strongly disagree 2—Disagree 3—Neutral 4—Agree 5—Strongly agree
Subjective norm (SN)/environmental factors	*SN1* (OPB1)—I know of others switching channels and get good outcome during search and purchase *SN2* (PSO1)—I start switching channels when I saw others switch between channels *SN3* (PSO2)—I will switch between channels during search and purchase if others advise me so *SN4* (PSO3)—I can be influenced by others when making decision	1—Strongly disagree 2—Disagree 3—Neutral 4—Agree 5—Strongly agree
Perceived behaviour control (PBC)/behavioral factors	*PBC1* (PSE1)—your carefulness level when searching for information and purchase a product *PBC2* (PSE2)—the time availability to help you switch between channels during search and purchase *PBC3* (PFC1)—the resources to help you switch between channels during search and purchase *PBC4* (PFC2)—yourself as a “smart customer”	1—Strongly disagree 2—Disagree 3—Neutral 4—Agree 5—Strongly agree
Likelihood of switching channel (INT)	What is the likelihood of you switching between channels when purchasing an electronic product?	1—Very unlikely 2—Unlikely 3—Neutral 4—Likely 5—Very likely

## Appendix B: Crosstabulation Results

Percentile Group of CLI * INT

**Table:**

			INT					
			1	2	3	4	5	Total
*Crosstab*								
Percentile group of CLI	1	Count	0	9	16	30	8	63
		% of total	0.0%	2.3%	4.1%	7.7%	2.1%	16.2%
	2	Count	1	5	12	42	12	72
		% of total	0.3%	1.3%	3.1%	10.8%	3.1%	18.5%
	3	Count	1	4	8	47	5	65
		% of total	0.3%	1.0%	2.1%	12.1%	1.3%	16.7%
	4	Count	0	3	16	96	19	133
		% of total	0.0%	0.8%	4.1%	24.4%	4.9%	34.1%
	5	Count	0	5	3	34	15	57
		% of total	0.0%	1.3%	0.8%	8.7%	3.8%	14.6%
Total		Count	2	26	55	248	59	390
		% of total	0.5%	6.7%	14.1%	63.6%	15.1%	100.0%

Percentile Group of CCS*INT

**Table:**

			INT					Total
			1	2	3	4	5	
*Crosstab*								
Percentile group of CCS	1	Count	1	7	9	18	2	37
		% of total	0.3%	1.8%	2.3%	4.6%	0.5%	9.5%
	2	Count	1	7	25	66	8	107
		% of total	0.3%	1.8%	6.4%	16.9%	2.1%	27.4%
	4	Count	0	9	19	141	35	204
		% of total	0.0%	2.3%	4.9%	36.2%	9.0%	52.3%
	5	Count	0	3	2	23	14	42
		% of total	0.0%	0.8%	0.5%	5.9%	3.6%	10.8%
Total		Count	2	26	55	248	59	390
		% of total	0.5%	6.7%	14.1%	63.6%	15.1%	100.0%

Percentile Group of ADM * INT

**Table:**

			INT					Total
			1	2	3	4	5	
*Crosstab*								
Percentile Group of ADM	1	Count	2	10	25	39	2	78
		% of total	0.5%	2.6%	6.4%	10.0%	0.5%	20.0%
	2	Count	0	3	11	35	6	55
		% of total	0.0%	0.8%	2.8%	9.0%	1.5%	14.1%
	3	Count	0	8	7	38	13	66
		% of total	0.0%	2.1%	1.8%	9.7%	3.3%	16.9%
	4	Count	0	2	6	81	14	103
		% of total	0.0%	0.5%	1.5%	20.8 %	3.6%	26.4%
	5	Count	0	3	6	55	24	88
		% of total	0.0%	0.8%	1.5%	14.1%	6.2%	22.6%
Total		Count	2	26	55	248	59	390
		% of total	0.5%	6.7%	14.1%	63.6%	15.1%	100.0%

Percentile Group of OPB * INT

**Table:**

			INT					Total
			1	2	3	4	5	
*Crosstab*								
Percentile Group of OPB	1	Count	1	16	33	58	14	122
		% of total	0.3%	4.1%	8.5%	14.9%	3.6%	31.3%
	3	Count	1	7	20	167	23	218
		% of total	0.3%	1.8%	5.1%	42.8%	5.9%	55.9%
	5	Count	0	3	2	23	22	50
		% of total	0.0%	0.8%	0.5%	5.9%	5.6%	12.8%
Total		Count	2	26	55	248	59	390
		% of total	0.5%	6.7%	14.1%	63.6%	15.1%	100.0%

Percentile Group of PSO * INT

**Table:**

			int					Total
			1	2	3	4	5	
*Crosstab*								
Percentile Group of PSO	1	Count	2	10	26	36	8	82
		% of total	0.5%	2.6%	6.7%	9.2%	2.1%	21.0%
	2	Count	0	1	7	44	4	56
		% of total	0.0%	0.3%	1.8%	11.3%	1.0%	14.4%
	2	Count	0	5	7	50	13	75
		% of total	0.0%	1.3%	1.8%	12.8%	3.3%	19.2%
	4	Count	0	8	12	88	16	124
		% of total	0.0%	2.1%	3.1%	22.6%	4.1%	31.8%
	5	Count	0	2	3	30	18	53
		% of total	0.0%	0.5%	0.8%	7.7%	4.6%	13.6%
Total		Count	2	26	55	248	59	390
		% of total	0.5%	6.7%	14.1%	63 6%	15.1%	100.0%

Percentile Group of PSE * INT

**Table:**

			INT					Total
			1	2	3	4	5	
*Crosstab*								
Percentile group of PSE	1	Count	2	19	26	46	11	104
		% of total	0.5%	4.9%	6.7%	11.8%	2.8%	26.7%
	2	Count	0	4	9	47	6	66
		% of total	0.0%	1.0%	2.3%	12.1%	1.5%	16.9%
	3	Count	0	2	13	93	15	123
		% of total	0.0%	0.5%	3.3%	23.8%	3.8%	31.5%
	5	Count	0	1	7	62	27	97
		% of total	0.0%	0.3%	1.8%	15.9%	6.9%	24.9%
Total		Count	2	26	55	248	59	390
		% of total	0.5%	6.7%	14.1%	63.6%	15.1%	100.0%

Percentile Group of PFC * INT

**Table:**

			int					Total
			1	2	3	4	5	
*Crosstab*								
Percentile Group of PFC	1	Count	0	10	5	25	6	46
		% of total	0.0%	2.6%	1.3%	6.4%	1.5%	11.8%
	2	Count	2	8	24	38	10	82
		% of total	0.5%	2.1%	6.2%	9.7%	2.6%	21.0%
	3	Count	0	3	9	45	5	62
		% of total	0.0%	0.8%	2.3%	11.5%	1.3%	15.9%
	4	Count	0	2	12	98	17	129
		% of total	0.0%	0.5%	3.1%	25.1%	4.4%	33.1%
	5	Count	0	3	5	42	21	71
		% of total	0.0%	0.8%	1.3%	10.8%	5.4%	18.2%
Total		Count	2	26	55	248	59	390
		% of total	0.5%	6.7%	14.1%	63.6%	15.1%	100.0%

## Appendix C: fsQCA Solutions

INT outcome

~ INT outcome

## Appendix D: fsQCA Solutions for the Subsample and Holdout samples

Subsample

Holdout sample
